# Inhibiting ferroptosis mitigates sheep sperm freezing damage

**DOI:** 10.3389/fvets.2025.1526474

**Published:** 2025-03-07

**Authors:** Erhan Hai, Boyuan Li, Yukun Song, Jian Zhang, Jiaxin Zhang

**Affiliations:** Inner Mongolia Key Laboratory of Sheep and Goat Genetics Breeding and Reproduction, College of Animal Science, Inner Mongolia Agricultural University, Hohhot, Inner Mongolia, China

**Keywords:** sheep sperm, cryopreservation, ferroptosis, apoptosis, ferroptosis inhibitors

## Abstract

**Objectives:**

To evaluate the roles of apoptosis and ferroptosis in cryopreservation-induced damage to sheep sperm, with a focus on assessing the effectiveness of inhibitors targeting these pathways.

**Methods:**

Initial analysis compared the expression of apoptotic marker Cleaved-caspase3 (CL-caspase3) and ferroptotic marker Transferrin receptor (TFRC) between fresh and cryopreserved sheep sperm. Elevated CL-caspase3 expression and sustained high TFRC expression post-cryopreservation suggested concurrent occurrence of apoptosis and ferroptosis. Consequently, the study employed Deferoxamine Mesylate (DFO), ferrostatin-1 (Fer-1), liproxstatin-1 (Lip-1), and the apoptosis inhibitor Z-VAD-FMK (Z-VAD) at concentrations ranging from 0 to 10 μM. Post-thaw assessments encompassed plasma membrane integrity, acrosome integrity, and ferroptosis biomarkers. Additional experiments were conducted to measure the expression of GPX4, a key regulator of ferroptosis.

**Results:**

Optimal concentrations (2 μM for DFO, Fer-1, and Lip-1; 5 μM for Z-VAD) significantly improved sperm motility and membrane integrity. Among these, Fer-1 demonstrated the greatest efficacy, reducing reactive oxygen species (ROS), lipid peroxidation, and Fe^2+^ levels. Z-VAD primarily decreased ROS but was less potent than ferroptosis inhibitors. Notably, Glutathione Peroxidase 4 (GPX4) expression was reduced post-cryopreservation, while Fer-1 supplementation restored its levels to those comparable with fresh sperm.

**Conclusion:**

Both apoptosis and ferroptosis play critical roles in sheep sperm cryopreservation. Fer-1 effectively enhanced cryopreservation outcomes by inhibiting ferroptosis, as evidenced by the restoration of GPX4 expression and improvement in sperm quality indicators. These findings highlight ferroptosis inhibition as a promising strategy for preserving genetic material, with implications for animal breeding and biodiversity conservation.

## 1 Introduction

Sperm cryopreservation damage includes extensive cell death and cryopreservation-induced capacitation (1). Increasing the number of viable cells after cryopreservation remains a primary challenge in the field (2). Cell death can be categorized into accidental cell death (ACD) and regulated cell death (RCD). ACD occurs due to physical damage to the cell plasma membrane beyond its self-regulatory capacity, resulting in cell death. In contrast, RCD is initiated by specific signal transduction pathways and can often be partially mitigated through pharmacological or genetic interventions (3).

ACD induced by intracellular ice crystal formation is widely recognized as the primary cause of cell death in thawed sperm. In sperm cryopreservation, effective management of dehydration and the use of osmoprotectants are essential strategies to mitigate this process (2, 4–6). Mathematical modeling and microscopic observation are utilized to detect intracellular ice crystal formation during cryopreservation (7). However, experimental studies on mammalian sperm cryopreserved with glycerol have yielded inconsistent findings compared to theoretical predictions (8–12). Glycerol undergoes hydration reactions with aqueous solvents, increasing the viscosity of intracellular fluid, thereby inhibiting ice crystal formation, which aids in cell protection (13). Morris et al. (14, 15) conducted successive observations using cryo-scanning electron microscopy and cryosubstitution on frozen semen from humans and horses, with cooling rates ranging from 0.3 to 3,000°C/min. Their findings consistently indicated the absence of intracellular ice crystal formation across all cooling rates, underscoring the pivotal role of glycerol.

Apoptosis serves as a critical indicator of sperm cryopreservation injury among various forms of RCD. Studies have identified apoptotic markers such as activation of the Caspase family, phosphatidylserine externalization, and mitochondrial membrane potential decrease during sperm cryopreservation (2, 16–18). Apoptosis can be initiated through both endogenous and exogenous pathways, ultimately resulting in the activation of Cleaved-Caspase3 (3). The addition of caspase inhibitors, such as Z-VAD-FMK (Z-VAD), effectively suppresses apoptosis (19). However, despite Z-VAD's efficacy in inhibiting apoptosis, its capacity to enhance the quality of thawed semen remains limited suggesting that apoptosis is not the sole form of RCD during sperm cryopreservation (2, 20, 21).

Lipid peroxidation is a crucial indicator of sperm cryopreservation injury, with ferroptosis, an extensively studied form of lipid peroxidation-induced RCD, being particularly significant (2, 22). Ferroptosis is a form of RCD characterized by iron-dependent lipid peroxidation reaching lethal levels, which involves the oxidation of polyunsaturated fatty acid-bound phospholipids (PUFA-PLs) on biological membranes (23). The regulation of redox state and iron levels forms the core framework of ferroptosis, distinguishing it from other forms of RCD and resulting in a necrotic phenotype (23). Although the term “ferroptosis” has not been widely applied in studies of sperm cryopreservation damage, substantial evidence suggests a strong association between ferroptosis and sperm cryopreservation injury (2). An increased proportion of plasma membrane PUFA-PLs is a hallmark of sperm maturation, crucial for maintaining membrane fluidity but also vulnerable to oxidation (24–26). Furthermore, oxidative stress is widely recognized as a primary cause of sperm cryopreservation damage (27). Consequently, ferroptosis is considered an inevitable consequence of sperm cryopreservation (2), although the effects of ferroptosis inhibitors on sperm cryopreservation remain poorly understood.

The primary objective of this study was to elucidate the role of ferroptosis in sperm freezing damage. To achieve this, Hu sheep were used as the subject animals, and we investigated the expression of the apoptotic marker CL-caspase3 and the ferroptosis marker TFRC in both fresh and frozen semen. Furthermore, we examined the effects of various inhibitors—including ferroptosis inhibitors (DFO, Fer-1, Lip-1) and the apoptosis inhibitor Z-VAD—on sperm quality parameters (total motility, progressive motility) and ferroptosis indicators (ROS, lipid peroxidation, Fe^2+^). Additionally, we assessed the expression of GPX4, a key regulator of ferroptosis, before and after cryopreservation, and evaluated the impact of Fer-1 on GPX4 expression. The findings of this study provide valuable insights into the mechanisms underlying sperm cryopreservation injury and suggest potential strategies for optimizing the cryopreservation process.

## 2 Materials and methods

### 2.1 Semen collection, cryopreservation, and thawing

Semen samples were obtained from five healthy 2-year-old sheep provided by Inner Mongolia Jin Lai Livestock Technology Co., Ltd. (Hohhot, China). The collection was performed using the artificial vagina method, with an estrous ewe serving as the stimulus for semen emission, these Hu sheep were bred once after reaching adulthood. Based on the ewe's lambing records and the growth status of the offspring, these Hu sheep are fertile. The sheep were maintained under standard management conditions; feeding occurred twice daily and water was available *ad libitum*. Sperm density was measured with a sperm density meter (IMV Technologies, France). For the computer-assisted sperm analysis system (CASA), physiological saline was used to dilute 2 μL of semen at room temperature, and 4 μL of the diluted sample was placed on a preheated Leja slide (025107-025108, IMV Technologies) and then in a CASA system (IVOS II, IMV Technologies) for sperm motility assessment. The temperature during the CASA analysis was maintained at 37°C. Samples with a volume of 1.5–2 mL, viability exceeding 75%, and sperm density above 3 × 10^8^/mL were selected for further processing at room temperature. Semen samples from each sheep collected on the same day (200 μL each) were then pooled together in a sterile container to form a combined sample, representing a single biological replicate for that individual sheep on that day. This process was repeated for each experimental day, ensuring each biological replicate consisted of semen from the same sheep on the same day.

The diluent formulation followed established protocols (28), including 1.8 g Tris, 1 g citric acid, 0.5 g glucose, 0.5 mL double antibiotics, 3 mL of 6% glycerol, 15 mL egg yolk, and ultrapure water to a final volume of 50 mL. The solution was filtered through a 0.22-μm filter and stored at 4°C. All reagents, except for egg yolk (Charoen Pokphand Group, China) and double antibiotics (15140122, Gibco, USA), were purchased from Sigma-Aldrich (USA).

The cryopreservation procedure comprised two main steps: semen dilution and equilibration, followed by freezing and thawing. Initially, the pooled semen was diluted to a sperm density of 2 × 10^8^ cells/mL using a standard semen diluent. Then, the diluted semen was placed into a 1,000 mL beaker filled with water at 30°C. Subsequently, the beaker containing the semen was put into a 4°C constant-temperature refrigerator and cooled from 30 to 4°C over 4 h following a controlled temperature curve validated in our previous study (28). Once at 4°C, the semen was aliquoted into straw using a pipette, sealed with sealing powder, and balanced on a fumigation rack for 2 h.

Subsequently, the fumigation rack was placed 4 cm above the liquid nitrogen surface in a polystyrene foam box (30 cm length × 20 cm width × 15 cm height; 2 cm wall thickness) pre-filled with liquid nitrogen. During this 7 min fumigation phase, the semen-filled straws were exposed to nitrogen vapor at −120°C (measured by infrared thermometer), allowing controlled ice crystal formation - a critical cryopreservation step (29). Immediately after fumigation, the straws were transferred from the foam box to a 50 L liquid nitrogen storage dewar (Cryosafe, China) for long-term preservation at −196°C. Thawing was performed after 7 days by immersion in a 37°C water bath for 30 s.

### 2.2 Addition of RCD inhibitors

Ferroptosis inhibitors, including DFO (CM00682, Proteintech, Wuhan, China), Lip-1 (CM04076, Proteintech), and Fer-1 (CM00719, Proteintech), as well as the apoptosis inhibitor Z-VAD (CM00937, Proteintech), were prepared in DMSO. Based on the manufacturers' recommendations and with the support of relevant research (30), these inhibitors were added to the semen diluent at final concentrations of 1, 2, 5, and 10 μM before the cryopreservation process. Control groups included C1 (no inhibitor) and C2 (0.1% DMSO). The final DMSO concentration in each treatment group was maintained at 0.1% of the total diluent volume.

### 2.3 Detection of total motility and progressive motility in thawed sheep sperm

The total motility (TM; %) and progressive motility (PM; %) of the thawed sheep sperm were assessed using a CASA system (IVOS II, IMV Technologies). For each sample, a minimum of five fields of view were analyzed, capturing at least 1,000 sperm cells per field. The optimal concentration of each inhibitor was determined based on the TM and PM values.

### 2.4 Detection of plasma membrane and acrosome integrity in thawed sheep spermatozoa

Plasma membrane integrity was assessed using Fixable Viability Dye eFluor™ 780 (FVD, 65-0865-14, Thermo Fisher Scientific, Bremen, Germany). Thawed semen samples (100 μL) were centrifuged at 300 × g for 5 min, the supernatant discarded, and the sperm washed with PBS. To this 499 μL of NC-BWW medium and 1 μL of 10 mM FVD were added. The mixture was incubated at 37°C for 10 min in the dark, then centrifuged and washed in PBS as above. The cells were resuspended in 200 μL of NC-BWW medium and analyzed using a CytoFLEX flow cytometer (Beckman Coulter, Brea, CA, USA). The flow cytometer used a 488-nm excitation light as the source for FVD; it used a 585/42 BP filter to collect signal intensity. The sample flow rate was maintained at 200–400 particles per second; at least 10,000 cells were analyzed in each sample.

Acrosome integrity was evaluated using a peanut agglutinin detection kit (PNA-FITC, Genemed Biotechnologies, San Francisco, CA, USA). Briefly, thawed semen was incubated at 37°C for 30 min, centrifuged at 300 × g for 5 min, and the supernatant was discarded. The sperm concentration was adjusted to 2 × 10^7^ sperm/mL with preservation solution. A 100-μL aliquot was mixed with 500-μL of clearing solution, centrifuged as above, washed with 200 μL of staining solution B, and incubated at room temperature for 20 min in the dark. After further centrifugation, 0.4 μL/mg of PI was added, incubated for 5 min at room temperature in the dark, and centrifuged again. The cells were resuspended in 1 mL of clearing solution and analyzed using the CytoFLEX flow cytometer. The flow cytometer used a 488-nm excitation light as the source for both PNA-FITC and PI. A 525/40 BP filter was used to collect the signal intensity of PNA-FITC; a 585/42 BP filter was utilized to collect the signal intensity of PI. The sample flow rate was maintained at 200–400 particles per second; at least 10,000 cells were analyzed in each sample. Single-stained tubes with PNA-FITC and PI were prepared for fluorescence compensation.

### 2.5 Detection of ROS levels

ROS levels in sperm were measured using a ROS kit (S0033S, Beyotime, Shanghai, China). For each group, 100 μL of thawed semen was centrifuged at 300 × g for 5 min. Supernatants were discarded, and pellets were washed with PBS. The sperm concentration was adjusted to 2 × 10^6^ sperm/mL using 499.5 μL of NC-BWW medium (containing 1 μL of 10 mM FVD) and 0.5 μL of 10 mM 2′,7′-dichlorodihydrofluorescein diacetate (DCFH-DA), and mixtures were incubated in the dark at 37°C for 10 min. Cells were washed as above and resuspended in 200 μL of NC-BWW medium and subjected to flow cytometric analysis. The flow cytometer used a 488-nm excitation light as the source for DCFH-DA. A 525/40 BP filter was used to collect the signal intensity of DCFH-DA. The sample flow rate was maintained at 200–400 particles per second; at least 10,000 cells were analyzed in each sample.

### 2.6 Detection of membrane lipid peroxidation levels

The procedure for detecting membrane lipid peroxidation levels closely followed that outlined in Section 2.6, with the key difference being the substitution of the DCFH-DA probe with 0.5 μL of 10 mM BODIPY-C11 (D3861, Thermo Fisher Scientific, Bremen, Germany), a specific fluorescent probe for lipid peroxidation. Like DCFH-DA, BODIPY-C11 was added to the sperm suspension and allowed to incubate at 37°C in the dark for 10 min. Following incubation and washing, the sperm were resuspended in NC-BWW medium (maintain constant sperm density) and analyzed using the CytoFLEX flow cytometer. The flow cytometer used a 488-nm excitation light as the source for oxidized C11-BODIPY (oxC11-BODIPY). A 525/40 BP filter was used to collect the signal intensity of oxC11-BODIPY. The sample flow rate was maintained at 200–400 particles per second; at least 10,000 cells were analyzed in each sample.

### 2.7 Detection of Fe^2+^ levels

For the detection of Fe^2+^ levels, thawed semen samples (100 μL) were washed and resuspended in NC-BWW medium (Adjust the sperm density to 2 × 10^∧^6 sperm/ml), following the same protocol as described in Sections 2.6, with the key difference being the substitution of the DCFH-DA probe with 1 μL of 1 mM FerroOrange (F374, DojinDo, Japan), was added to the sperm suspension. The samples were then incubated at 37°C in the dark for 30 min to allow the probe to bind to Fe^2+^ ions within the sperm cells. After incubation, the sperm were washed to remove unbound probe and resuspended in 200 μL of NC-BWW medium (maintain constant sperm density). The final suspension was analyzed using the CytoFLEX flow cytometer. The flow cytometer used a 488-nm excitation light as the source for Fe^2+^. A 585/42 BP filter was used to collect the signal intensity of Fe^2+^. The sample flow rate was maintained at 200–400 particles per second; at least 10,000 cells were analyzed in each sample.

### 2.8 Flow cytometric analysis of Cl-caspase3, TFRC and GPX4 proteins

Flow cytometry was employed to analyze intracellular protein expression in sperm cells, comparing fresh sperm with frozen-thawed sperm. Flow cytometry intracellular fixation and permeabilization buffers were obtained from Thermo Fisher Scientific (88-8824-00). The primary antibodies included rabbit polyclonal IgG isotype control (30000-0-AP, Proteintech), mouse monoclonal IgG2α isotype control (61,656, Cell Signaling Technology, Danvers, MA, USA), Transferrin receptor (TFRC, 66180-1-lg, Proteintech), CL-caspase3 (9,661, Cell Signaling Technology), and glutathione peroxidase 4 (GPX4, 30388-1-AP, Proteintech). The isotype control for TFRC was mouse monoclonal IgG2α isotype control, whereas the isotype controls for CL-caspase3 and GPX4 were rabbit polyclonal IgG isotype controls. The secondary antibodies included CoraLite488-conjugated donkey anti-rabbit IgG (SA00013-6, Proteintech) and donkey anti-mouse IgG (SA00013-5, Proteintech).

Cells were washed with PBS and subjected to fixation and permeabilization steps. Specifically, 200 μL of fixation buffer was added to the cells following incubation in the dark at room temperature for 30 min. Next, 300 μL of permeabilization buffer was added, and samples were centrifuged at 300 × g for 5 min. This step was repeated twice, and then 400 μL of permeabilization buffer was added, followed by incubation in the dark at room temperature for 15 min. Following centrifugation, cells were blocked in 100 μL of permeabilization buffer and 400 μL of BSA (10%) in the dark at room temperature for 30 min.

After blocking, washed cells were incubated with primary antibodies (final concentration of 5 μg/mL) in 200 μL of permeabilization buffer for 60 min in the dark at room temperature. Cells were then washed with permeabilization buffer, followed by incubation with secondary antibodies (1:200) in 200 μL of permeabilization buffer for 30 min in the dark at room temperature. Following additional washes, cells were resuspended in 200 μL of NC-BWW medium.

Samples were analyzed using flow cytometry with a rigorous protocol that ensured the detection of 10,000 cells per group. During this process, the expression level of each protein was determined by measuring the intensity above its respective isotype control. All target proteins were assayed via the flow cytometer used a 488-nm excitation light as the source for CoraLite488. A 525/40 BP filter was used to collect the signal intensity of CoraLite488. FlowJo (v10.8.1) software was utilized to visualize and analyze the flow cytometry data.

### 2.9 Statistical analyses

For the flow cytometric analysis of proteins, statistical analysis was conducted using a two-tailed *t*-test within GraphPad Prism (version 10.1.0) software. Conversely, all remaining experiments were statistically analyzed using a one-way ANOVA (Analysis of Variance) within SPSS (v22.0) software. In both cases, a *P* < 0.05 was deemed significant, and data are presented as the mean ± SEM. Additionally, FLOW JO software (v 10.8.1) was relied upon for the analysis of flow cytometry data.

## 3 Results

### 3.1 Apoptosis and ferroptosis may occur simultaneously during sperm cryopreservation

**Figure 1:**
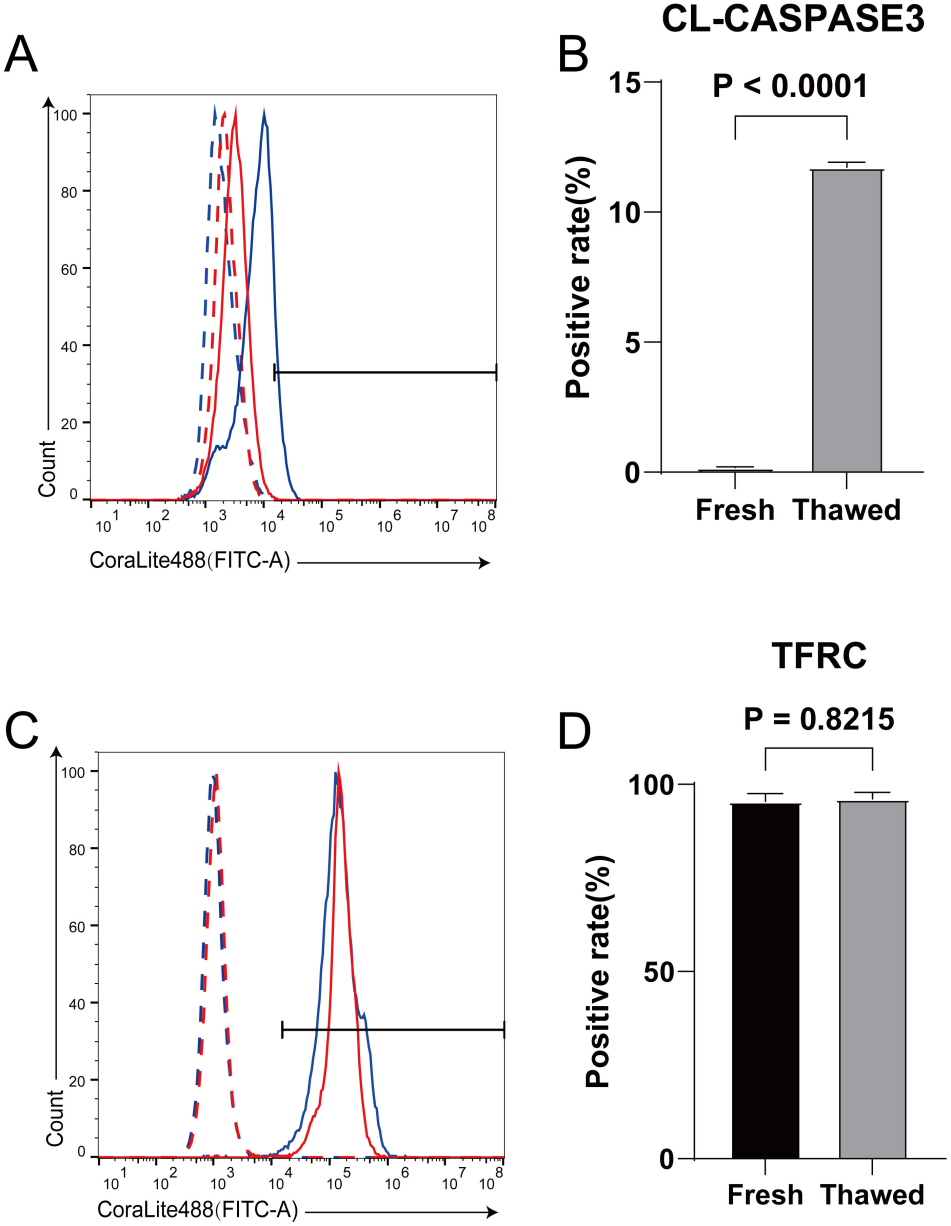
Detection of expression levels of CL-caspase3 and TFRC in fresh and frozen-thawed sperm using flow cytometry. **(A)** Expression levels of CL-caspase3 proteins in fresh sperm and frozen-thawed sperm. The dashed line represents the isotype control, while the solid line represents the target protein. The red color indicates the fresh sperm group, and the blue color indicates the frozen-thawed sperm group. **(B)** The statistical result graph of **(A)**. **(C)** Expression levels of TFRC proteins in fresh sperm and frozen-thawed sperm. The dashed line represents the isotype control, while the solid line represents the target protein. The red color indicates the fresh sperm group, and the blue color indicates the frozen-thawed sperm group. **(D)** The statistical result graph of **(C)**. The proportion of the gated region in flow cytometry represents the expression level of the protein, and the expression level of each protein was determined by measuring the intensity above its respective isotype control. *n* = 3.

The expression level and positive rate of CL-caspase3 can directly reflect the apoptosis level of cells (3), while the expression level of TFRC is positively correlated with the sensitivity of cells to ferroptosis (31). In our study, we found that the expression level of Cl-caspase3 in thawed sperm is significantly higher than that in Fresh sperm, indicating that apoptosis may have occurred (Figures 1A, B). On the other hand, although there is no significant difference in TFRC expression between thawed and Fresh sperm, both show extremely high positive rates (Figures 1C, D), suggesting that sperm are susceptible to ferroptosis.

### 3.2 The role of regulated cell death inhibitors in sperm cryopreservation

#### 3.2.1 Determination of optimal concentrations of RCD inhibitors

The rescue level of inhibitors on cell death can indicate whether various forms of cell death occur in cells and the proportion of their occurrence (3). Optimal concentrations of RCD inhibitors were determined based on the TM and PM of thawed spermatozoa (Table 1). The 2 μM concentrations of DFO, Fer-1, and Lip significantly enhanced both TM and PM compared to control groups (C1 and C2). Fer-1 (2 μM) exhibited the highest TM and PM improvement, while 5 μM Z-VAD significantly increased TM without affecting PM compared to controls. Therefore, concentrations of 2 μM for DFO, Fer-1, and Lip-1, and 5 μM for Z-VAD were selected for subsequent experiments.

**Table 1:** Total motility and progressive motility of thawed spermatozoa treated with regulated cell death inhibitors.

**Treatment**	**Total motility (%)**	**Progressive motility (%)**
Control (C1)	50.9 ± 7.24^h^	29.4 ± 5.36^h^
Control (C2)	54.78 ± 3.17^gh^	27.94 ± 2.11^h^
DFO (1 μM)	62.84 ± 2.33^de^	42.86 ± 4.18^bcd^
DFO (2 μM)	70.40 ± 1.81^bc^	44.94 ± 2.58^bc^
DFO (5 μM)	65.70 ± 2.61^cd^	40.34 ± 2.98^cde^
DFO (10 μM)	55.78 ± 4.64^fgh^	34.58 ± 6.44^efg^
Fer-1 (1 μM)	67.88 ± 1.45^bcd^	47.38 ± 5.27^b^
Fer-1 (2 μM)	75.88 ± 2.21^a^	60.76 ± 2.64^a^
Fer-1 (5 μM)	69.26 ± 2.84^bc^	36.1 ± 5.18^efg^
Fer-1 (10 μM)	66.66 ± 2.25^bcd^	46.74 ± 5.71^b^
Lip-1 (1 μM)	60.16 ± 4.49^ef^	33.96 ± 3.32^fg^
Lip-1 (2 μM)	71.62 ± 1.82^ab^	45.78 ± 1.02^bc^
Lip-1 (5 μM)	71.44 ± 2.19^ab^	40.38 ± 4.09^cde^
Lip-1 (10 μM)	63.16 ± 0.68^de^	36.8 ± 3.31^efg^
Z-VAD (1 μM)	51.98 ± 3.18^h^	34.02 ± 2.96^fg^
Z-VAD (2 μM)	57.12 ± 1.62^fg^	31.73 ± 4.45^gh^
Z-VAD (5 μM)	63.33 ± 2.66^de^	38.12 ± 2.90^def^
Z-VAD (10 μM)	63.7 ± 4.39^de^	31.78 ± 3.60^gh^

Significant differences are indicated by different letters (*P* < 0.05). *n* = 10.

#### 3.2.2 Ferroptosis inhibitors significantly improve membrane structural integrity in thawed sperm

**Figure 2:**
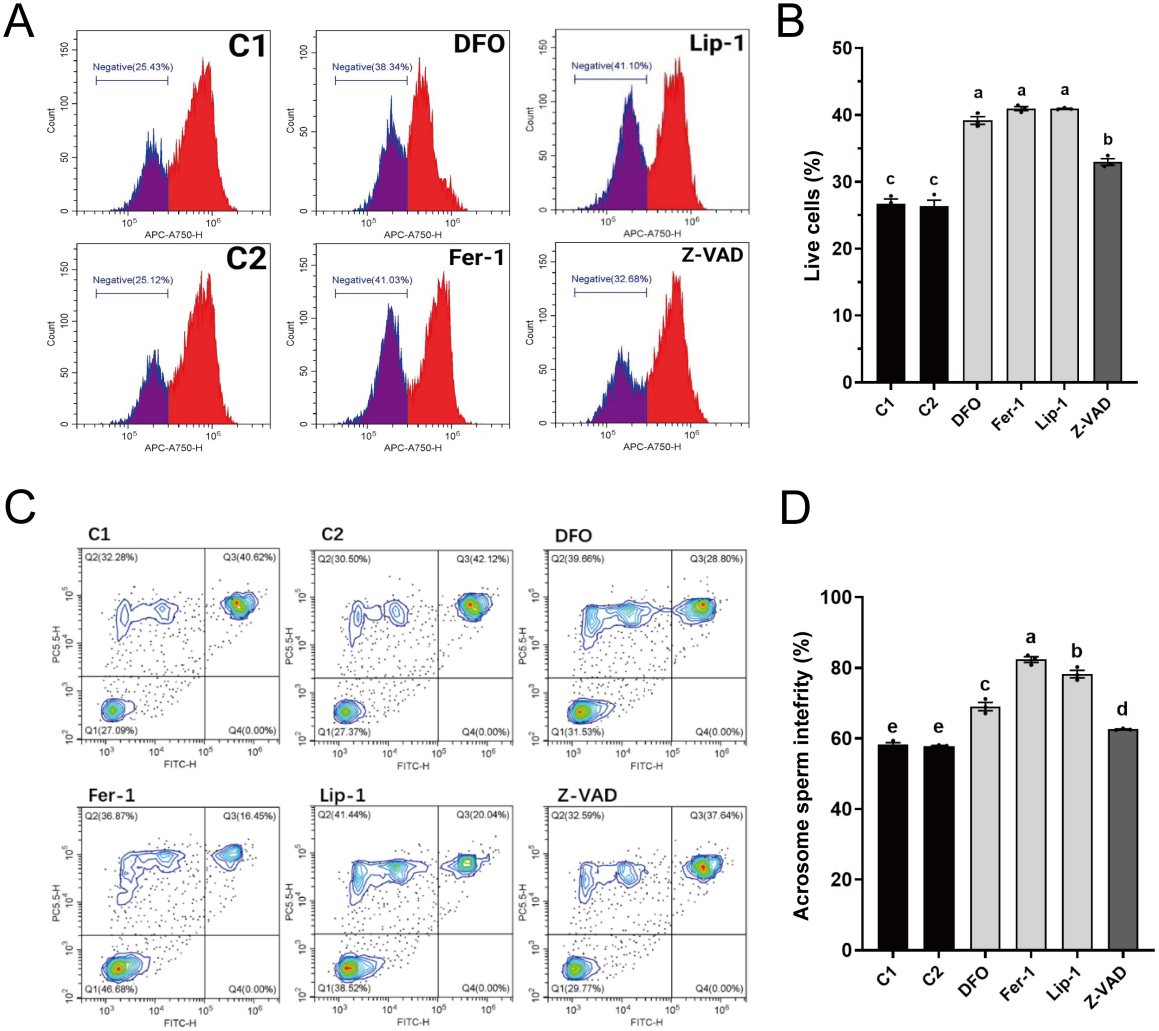
Effects of different regulated cell death inhibitors on the membrane structural integrity of thawed spermatozoa. **(A)** Flow cytometry results showing plasma membrane integrity. The gated cell population represents spermatozoa with intact plasma membranes. **(B)** Statistical graph depicting the percentage of spermatozoa with intact plasma membranes from **(A)**. **(C)** Flow cytometry results showing acrosome integrity. Quadrant Q1: intact plasma membranes and acrosomes; Q2: damaged plasma membranes but intact acrosomes; Q3: damaged plasma membranes and acrosomes; Q4: intact plasma membranes but damaged acrosomes. **(D)** Statistical graph of the combined percentages of cells with intact plasma membranes (Q1 + Q2) from **(C)**. Different letters in **(B, D)** indicate significant differences (*P* < 0.05). *n* = 3.

The integrity of the plasma membrane and acrosome is crucial for evaluating the quality of thawed spermatozoa. Plasma membrane integrity analysis (Figures 2A, B) showed that all RCD inhibitors significantly enhanced plasma membrane integrity compared to control groups C1 (26.74 ± 1.01%) and C2 (26.37 ± 1.26%). Notably, the ferroptosis inhibitors (DFO: 39.19 ± 0.81%, Fer-1: 40.96 ± 0.42%, and Lip-1: 40.94 ± 0.11%) exhibited significantly higher percentages of intact plasma membranes than Z-VAD (32.97 ± 0.69%), with Fer-1 achieving the highest percentage.

Analysis of acrosome integrity (Figures 2C, D) revealed that all RCD inhibitors significantly improved acrosome integrity compared to C1 (58.20 ± 0.82%) and C2 (57.83 ± 0.25%). Again, the ferroptosis inhibitors (DFO: 69.04 ± 1.67%, Fer-1: 82.37 ± 1.14%, and Lip-1: 78.24 ± 1.52%) exhibited significantly higher percentages of intact acrosomes than Z-VAD (62.56 ± 0.19%), with Fer-1 exhibiting the highest percentage.

#### 3.2.3 Mitigation of ferroptosis in thawed sperm by RCD inhibitors

Given the critical role of RCD inhibitors in identifying cell death types, we hypothesized that ferroptosis predominates during sheep sperm cryopreservation. Therefore, we investigated the impact of various RCD inhibitors on key ferroptosis indicators in thawed spermatozoa, including ROS, lipid peroxidation, and Fe^2+^ levels.

**Figure 3:**
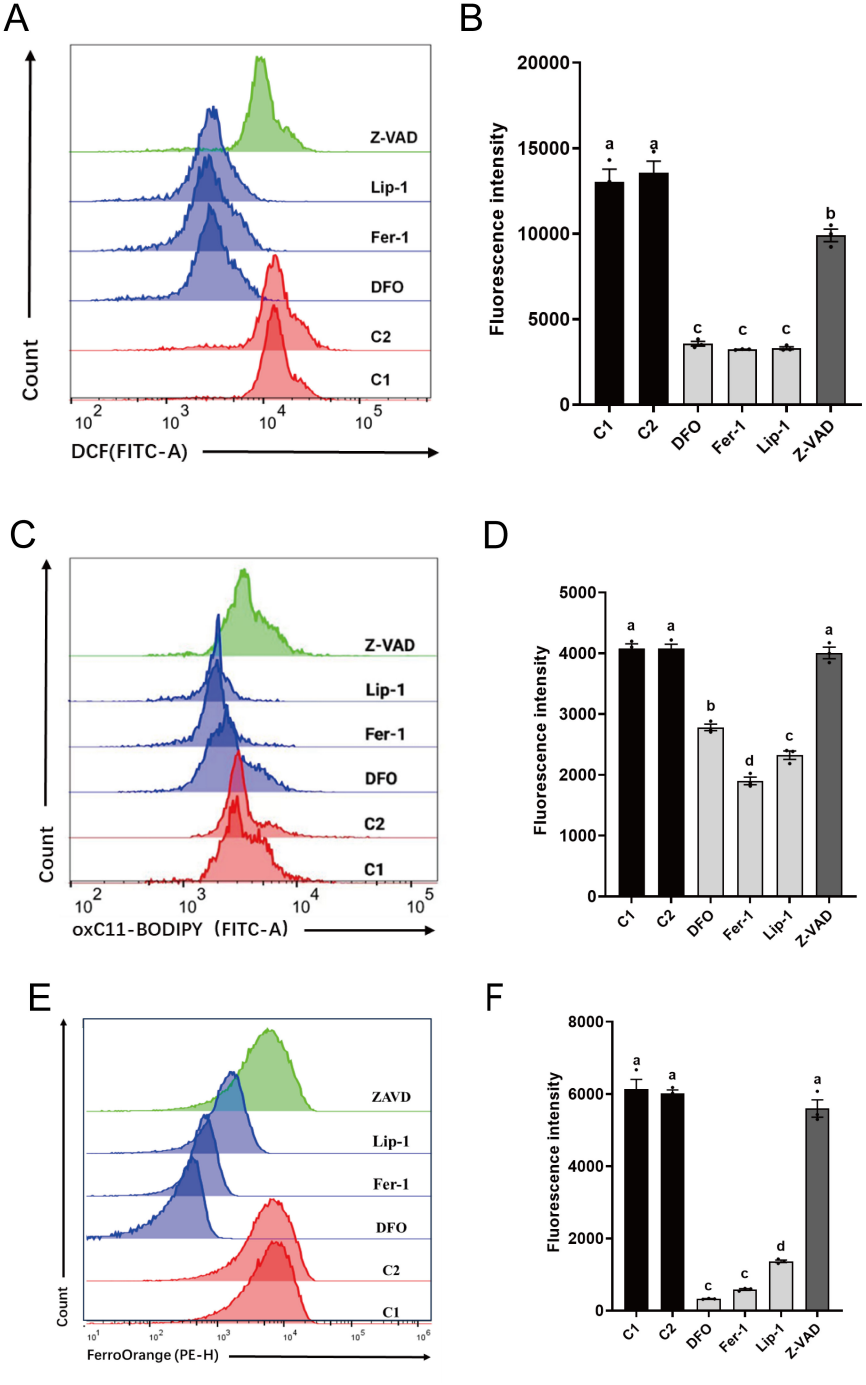
Effects of different regulated cell death inhibitors on ferroptosis in thawed spermatozoa. **(A)** Flow cytometry results showing ROS levels. **(B)** Statistical graph illustrating ROS levels **(A)**. **(C)** Flow cytometry results depicting lipid peroxidation levels. **(D)** Statistical graph presenting lipid peroxidation levels **(C)**. **(E)** Flow cytometry results indicating Fe^2+^ levels. **(F)** Statistical graph displaying Fe^2+^ levels **(E)**. Different letters in (**B**, **D**, and **F**) indicate significant differences (*P* < 0.05). *n* = 3.

Results from ROS analysis (Figures 3A, B) showed that all RCD inhibitors significantly reduced ROS levels in spermatozoa compared to C1 (13030.86 ± 1046.54) and C2 (13561.03 ± 967.76). Ferroptosis inhibitors (DFO: 3560.83 ± 195.82, Fer-1: 3,229 ± 22.87, and Lip-1: 3300.46 ± 121.75) exhibited significantly lower ROS levels than Z-VAD (9903.63 ± 518.64).

Analysis of lipid peroxidation levels (Figures 3C, D) revealed that spermatozoa treated with ferroptosis inhibitors (DFO: 2781.90 ± 75.34, Fer-1: 1900.70 ± 89.80, and Lip-1: 2323.23 ± 97.57) also had significantly lower lipid peroxide levels compared to C1 (4076.33 ± 111.59) and C2 (4079.10 ± 99.38), as well as Z-VAD (4006.13 ± 134.02). No significant differences were observed between Z-VAD and C1 or C2.

Analysis of Fe^2+^ levels (Figures 3E, F) indicated that spermatozoa treated with ferroptosis inhibitors (DFO: 326.33 ± 10.33, Fer-1: 585.67 ± 32.86, and Lip-1: 1365.67 ± 50.84) exhibited significantly lower Fe^2+^ levels compared to C1 (6,138 ± 376.89) and C2 (6,018 ± 132.69), as well as Z-VAD (5598.67 ± 340.53). No significant differences were observed between Z-VAD and C1 or C2. In summary, these findings highlight the effectiveness of ferroptosis inhibitors over the apoptosis inhibitor in reducing oxidative stress, lipid peroxidation, and Fe^2+^ levels in cryopreserved spermatozoa.

### 3.3 Fer-1 can increase the expression level of GPX4 in frozen-thawed sperm

**Figure 4:**
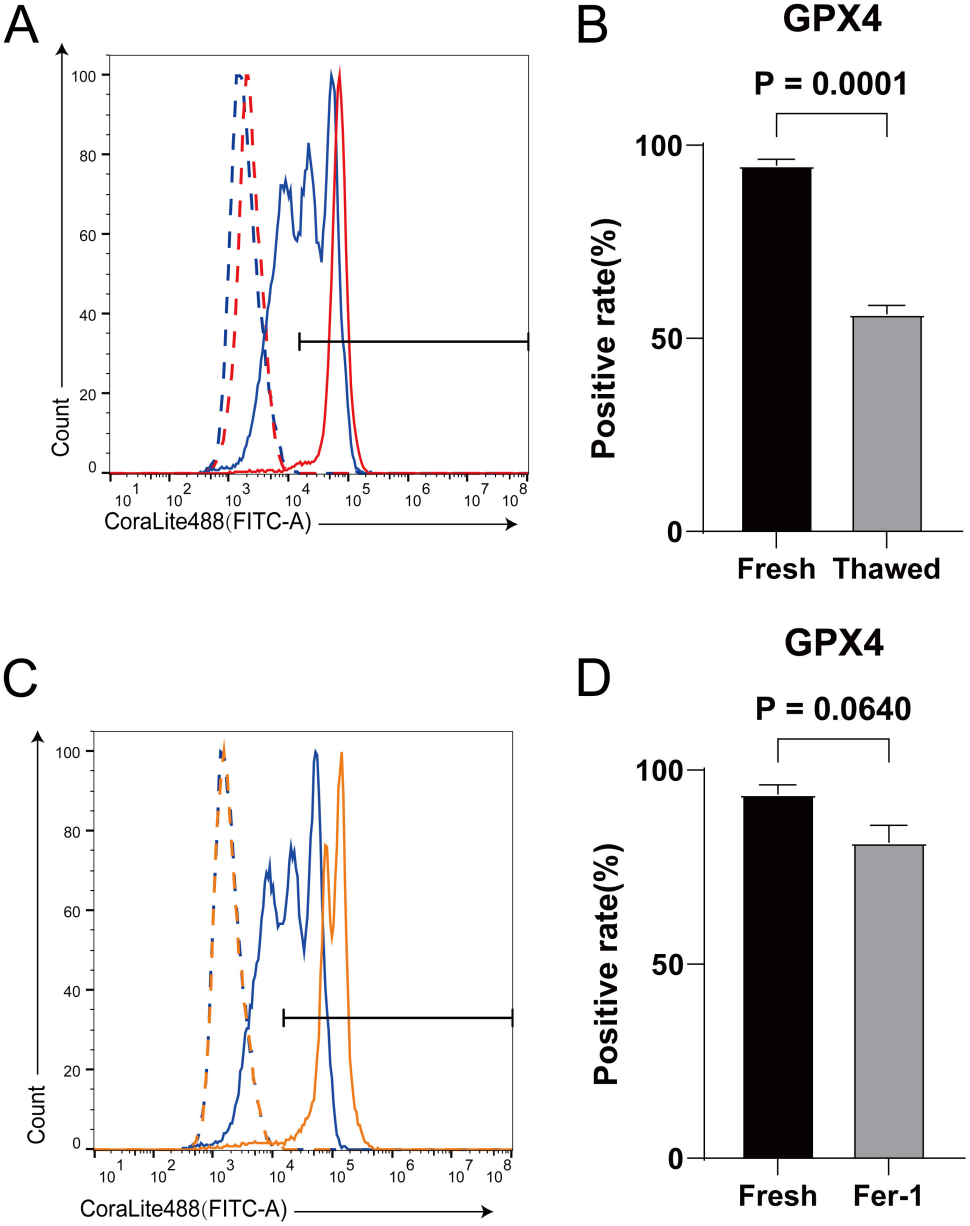
The effect of Fer-1 on the expression level of GPX4 protein in frozen-thawed sperm. **(A)** Expression levels of GPX4 proteins in fresh sperm and frozen-thawed sperm. The dashed line represents the isotype control, while the solid line represents the target protein. The red color indicates the fresh sperm group, and the blue color indicates the frozen-thawed sperm group. **(B)** The statistical result graph of **(A)**. **(C)** Expression levels of GPX4 proteins in fresh sperm and frozen-thawed sperm after addition of Fer-1. The dashed line represents the isotype control, while the solid line represents the target protein. The orange color indicates the fresh sperm group, and the blue color indicates the frozen-thawed sperm group. **(D)** The statistical result graph of **(C)**. The proportion of the gated region in flow cytometry represents the expression level of the protein, and the expression level of each protein was determined by measuring the intensity above its respective isotype control. *n* = 3.

GPX4 is a key protein in ferroptosis (32). Inhibiting the expression level of GPX4 can lead to the occurrence of ferroptosis in cells (23). The expression level of GPX4 in sperm significantly decreases after cryopreservation (Figures 4A, B). However, after adding Fer-1, its expression level is not significantly different from that in fresh sperms (Figures 4C, D). It indicates that the occurrence of ferroptosis during sheep sperm cryopreservation may be triggered by the decreased expression of GPX4.

## 4 Discussion

This study demonstrates that supplementing the diluent with ferroptosis inhibitors significantly enhances the quality of thawed sperm. This improvement is likely attributed to ferroptosis emerging as the primary form of RCD affecting sperm during cryopreservation, overshadowing the traditional focus on apoptosis. Historically, research has predominantly emphasized apoptosis while neglecting other potential RCD mechanisms in sperm cryopreservation. This oversight may have impeded advancements in sperm cryopreservation technology (2).

Oxidative stress is the most significant factor contributing to sperm cryopreservation damage (27). During the freezing and thawing stages, extreme changes in the extracellular microenvironment, coupled with the sperm's inherent lack of antioxidant capacity–due to the absence of cytoplasmic antioxidant molecules and the inability to mount a genomic antioxidant response–can easily lead to a rapid increase in ROS levels, resulting in cell death (2). Oxidative stress can induce various types of RCD, among which ferroptosis, driven by Fe^2+^-induced lipid peroxidation through the Fenton reaction, has garnered significant attention in recent years (23). However, research on ferroptosis in sperm cryopreservation has not been reported.

Lipid peroxidation is not only a key indicator of sperm cryopreservation injury but also a specific marker that distinguishes ferroptosis from other types of RCD (2). The primary source of ROS during sperm cryopreservation is likely electron leakage from the mitochondrial electron transport chain. Interestingly, lipid peroxidation production is thought to be associated with the Fenton reaction (2, 24, 26, 33–35). Although changes in Fe^2+^ levels during sperm cryopreservation remain unclear, this study demonstrates that ferroptosis inhibitors (DFO, Lip-1, and Fer-1) can reduce Fe^2+^ levels in thawed sperm, indicating elevated Fe^2+^ levels during this process. Given that TFRC is a primary protein involved in intracellular iron regulation, cells exhibiting high levels of TFRC expression are particularly susceptible to ferroptosis. Consequently, despite the fact that TFRC expression may not change significantly following cryopreservation of sperm (2, 23, 31), the inherently high expression of TFRC in sheep sperm could potentially contribute to the accumulation of iron ions after cryopreservation. Furthermore, the differential expression of ferritin heavy chain 1 (FTH1) in thawed sperm of dairy goats with varying freezing resistances underscores the importance of iron regulation in sperm cryopreservation injury (36).

Significant differences in freezing resistance among thawed semen samples from different individuals have prompted numerous studies to utilize omics analyses and other methods to elucidate this biological phenomenon (37). This variability may be linked to the sperm's ability to resist ferroptosis, as evidenced by the iron death redox regulatory network and the previously mentioned FTH1 (2). Glutathione peroxidase 4 (GPX4), a core protein involved in ferroptosis, has been highlighted in this context (32). Previous research has demonstrated that measuring GPX4 expression at the mRNA and protein levels in fresh sperm can accurately predict sperm freezing resistance (38). This is because GPX4 plays a crucial role in reducing lipid peroxides after sperm thawing by utilizing glutathione (GSH) (39). In the present study, it was found that the downregulation of GPX4 after sperm cryopreservation may be an important factor contributing to sperm freezing damage.

Small molecule compounds that effectively inhibit ferroptosis generally fall into two major categories: lipophilic reducing agents (such as Lip-1) and iron chelators (such as DFO). Fer-1 stands out due to its combined properties of lipophilic reduction and iron chelation (30). As research on ferroptosis progresses, specific lipophilic reducing agents widely used in sperm cryopreservation studies, such as α-tocopherol (α-TOH) and Trolox, have emerged as potent ferroptosis inhibitors (2, 30). α-TOH significantly improves the biochemical and kinetic properties of frozen-preserved boar semen (40), while reducing lipid peroxidation and maintaining sperm viability in chilled (5°C) horse sperm (41). Trolox, on the other hand, protects plasma membrane integrity and mitochondrial structure in frozen goat semen (42), ensuring high-quality thawed semen in both healthy individuals and patients with oligozoospermia (43).

The integration of ferroptosis inhibitors into sperm cryopreservation represents a promising strategy to enhance the quality of cryopreserved sperm. This study sheds light on ferroptosis as a critical mechanism of cell death during sperm freezing and thawing, highlighting its potential to surpass traditional apoptosis-centric approaches. Further elucidating the roles of key molecules like GPX4 in ferroptosis resistance could lead to targeted interventions to mitigate oxidative damage and improve sperm survival post-thaw. Future research directions should explore these mechanisms in greater detail, aiming to refine cryopreservation techniques and ultimately benefit infertility treatments and livestock breeding programs.

## 5 Conclusion

This study highlights the effectiveness of ferroptosis inhibitors, including DFO, Lip-1, and Fer-1, in mitigating oxidative damage during sperm cryopreservation, thus offering promising strategies to enhance the quality and viability of cryopreserved sperm in animal breeding. By targeting ferroptosis, these inhibitors address a critical pathway contributing to oxidative damage during freezing and thawing processes. This shift in focus from apoptosis to ferroptosis underscores significant implications for improving sperm preservation techniques in animal reproduction. Future research should continue to elucidate the mechanisms of ferroptosis in sperm preservation, paving the way for advancements in reproductive biotechnologies and enhanced breeding programs. Understanding these pathways could lead to the discovery of novel therapeutic targets and strategies to minimize oxidative stress and improve outcomes in animal husbandry.

## Data Availability

The original contributions presented in the study are included in the article/supplementary material, further inquiries can be directed to the corresponding author.
